# On a Common Misconception Regarding the de Broglie–Bohm Theory

**DOI:** 10.3390/e20060440

**Published:** 2018-06-05

**Authors:** Oliver Passon

**Affiliations:** School for Mathematics and Natural Sciences, University of Wuppertal, Gaußstr. 20, 42119 Wuppertal, Germany; passon@uni-wuppertal.de; Tel.: +49-202-439-2490

**Keywords:** quantum theory, de Broglie–Bohm theory, contextuality

## Abstract

We discuss a common misconception regarding the de Broglie–Bohm (dBB) theory; namely, that it not only assigns a position to each quantum object but also contains the momenta as “hidden variables”. Sometimes this alleged property of the theory is even used to argue that the dBB theory is inconsistent with quantum theory. We explain why this claim is unfounded and show in particular how this misconception veils the true novelty of the dBB theory.

## 1. Introduction

Bohm’s interpretation of quantum theory [1] is an example for a (non-local) hidden variable theory which avoids the notorious measurement problem of quantum mechanics. It is usually assumed that this theory reproduces all predictions of ordinary quantum theory. Some times this claim is qualified by the remark “as long as the latter are unambiguous” (see e.g., [2]). This refers e.g., to the fact that the quantum mechanical predictions regarding “time” (e.g., the “arrival time” or the “tunneling time”) are problematic.

However, there is a common misconception with regard to the question which quantities exactly are promoted to the “hidden variable” status. For example Mario Bunge writes ([3], p. 453):

“In particular, Bohm enriched standard non-relativistic quantum mechanics with a classical position coordinate and the corresponding momentum [...].”

While it is true that Bohmian mechanics assigns a well defined position to each quantum object at any moment, this quote suggests, that each particle possesses also a well defined momentum. Given that a well defined position apparently translates into a velocity which may be multiplied with the mass of the corresponding object (say, the electron) this claim is seemingly very natural. However, it turns out to be wrong nevertheless. At the same time this alleged property of Bohmian mechanics has been used to argue that this theory is inconsistent with quantum theory. An argument along these lines was recently given by Michael Nauenberg [4]. We think that the explanation why this charge is unfounded provides a good opportunity to combat certain long-standing prejudices surrounding the Bohm interpretation. In addition it provides a opportunity to unveil what we take to be the true novelty of the Bohm theory.

In Section 2 we will briefly outline the basics of the Bohm theory. Section 3 contains the most recent example for the criticism outlined above, namely the interesting contribution of Michael Nauenberg [4]. Section 4 explains the underlying misconception on which these claims are based. Finally, we will summarize our discussion in Section 5.

## 2. A (Very) Brief Introduction to the dBB Theory

In Bohm’s interpretation a system of *N* particles is described by the wave function (i.e., the solution of the corresponding Schrödinger equation) and the configuration $q_{k}$, i.e., the actual positions of the quantum objects. Thus, Bohm has to add an “equation of motion” or “guiding equation” for the positions to the formalism. Assuming a wave function, ψ=Rexp(iS/ℏ), the guiding equation for the position *q* of a spin-less particle takes (in the 1-particle case) the form:

(1) $$\begin{array}{c} \frac{dq}{dt}=\frac{\vec{\nabla }S}{m}. \end{array}$$

Note, that this equation is of first order in time, i.e., the initial configuration alone fixes the motion uniquely. The generalization to the many particle case and including spin is straightforward [5]. Choosing initial conditions for the position according to Born’s rule (i.e., $\rho ={\left|\psi \right|}^{2}$) the continuity equation

(2) $$\begin{array}{c} \frac{d\rho }{dt}+\nabla j=0 \end{array}$$

with the usual quantum mechanical probability current

(3) $$j=\frac{\hbar }{2mi}\left[\psi^{*}\left(\nabla \psi \right)-\left(\nabla \psi^{*}\right)\psi \right]$$

(4) $$=\rho \frac{\nabla S}{m}$$

ensures that the positions remain ${\left|\psi \right|}^{2}$ distributed. Hence, in terms of position any measurement yields exactly the result of the standard formalism. Thus, the guidance equation is consistent with the requirements of quantum mechanics. An other way to bring out the difference between ordinary QM and the dBB theory is the following: In QM the probability current refers to the probability to measure a certain position. Within the dBB theory it can be viewed as the probability of the particle to be at a certain position—independent of any measurement. The different strategies to explain why the ${\left|\psi \right|}^{2}$ distribution holds initially are critically examined by Norsen [6].

We may add a remark with respect to further references which provide a full exposition. Bohm’s work was essentially an independent re-discovery of work that was done by Louis de Broglie already in the 1920s [7]. Thus the term “de Broglie–Bohm” (dBB) theory is more appropriate. Further more there are different schools of the dBB theory. While [5] presents a version called “Bohmian mechanics”, the books by Bohm and Hiley or Holland [8,9] stick closer to the original presentation of Bohm from 1952 [1]; sometimes called “ontological” or “causal” interpretation of quantum mechanics. However, these distinctions play a minor role in what follows.

## 3. The Asymmetry between Position and Momentum

We now turn to the criticism mentioned above, which arises if the special role of the position observable within the de Broglie–Bohm theory is not taken into account. Reference [4] claims that the equivalence holds only in the “coordinate representation” while moving into the “momentum representation” leads to conflicting results. To reach this conclusion it introduces the “velocity operator” (i.e., the momentum operator divided by the mass):

(5) $$\begin{array}{c} \vec{v}=-\frac{i\hbar }{m}{\vec{\nabla }}_{q}. \end{array}$$

The author investigates the expectation value of this operator (in particular its second moment) and finds a result which differs from the second moment of the expression $v=\frac{\nabla S}{m}$, i.e., the velocity of the Bohmian particles (called ${\vec{v}}_{B}$ by this author). In [4] it is further claimed that this feature has gone unnoticed by the recent literature on the Bohm interpretation and we will come to this point shortly.

Reference [4] discusses as a specific example the velocity of a Bohmian particle for stationary solutions with vanishing phase, i.e., described by real wave functions. Such wave functions describe for example the electron in the ground state of the hydrogen or the energy eigenstates of the quantum mechanical harmonic oscillator. In all these cases the velocity vanishes ($v=\frac{\nabla S}{m}=0$). Examples like this are among the oldest objections raised against the Bohm interpretation [10]. Already in 1953 Einstein discussed a particle-in-a-box example with vanishing velocity. Clearly, that these examples are old calls into question the originality of this argument but as such not its soundness. Interestingly, Einstein did not conclude that the Bohm interpretation is in contradiction with quantum mechanics but finds fault with it since [11]:

“The vanishing of the velocity contradicts the well-founded requirement, that in the case of a macro-system the motion should agree approximately with the motion following from classical mechanics.”

Now, Reference [4] goes a step further and claims the refutation of Bohm’s interpretation:

“But this result contradicts the fact that in quantum mechanics the velocity or momentum distribution for stationary solutions, given by the absolute square of the Fourier transform of ψ in coordinate space, is not a delta function at $\vec{v}=0$, as is implied by Bohm’s interpretation.” (p. 44)

Before we turn to the question why this statement is unfounded we may note that along similar lines the argument could have been developed further. Comparing the expectation values for the kinetic energy with the expression $\langle \frac{mv_{B}^{2}}{2}\rangle$ or the angular momentum with the alleged Bohmian prediction “$\vec{L}=\vec{r}\times m{\vec{v}}_{B}$” would have yielded a host of “predictions” which differ from the corresponding quantum mechanical result. In addition, perhaps most devastating: given a momentum $\vec{p}=m{\vec{v}}_{B}$ would in general contradict with Heisenberg’s uncertainty principle.

## 4. Contextuality of All Observables Other than Position

Now, we finally should explain why and where all this reasoning goes astray. In Reference [4] it is apparently presumed that in Bohm’s theory the expression “$\vec{p}=m{\vec{v}}_{B}$” should describe a momentum and should relate to the momentum operator of standard QM. After having shown that this does not work it is claimed that:

“[...] this interpretation is not only inconsistent with the standard formulation of quantum mechanics, but also with classical mechanics, where momentum is defined by the relation $\vec{p}=m\vec{v}$.” (p. 45)

Let us discuss these points in reversed order. Bohm’s interpretation is certainly inconsistent with classical mechanics since it is a quantum theory. In the case of Bohmian mechanics there can be something added to this general argument. Given that the guiding equation is of first order one would not even expect that concepts of (second order) Newtonian mechanics (like momentum and work) play any role on the level of individual particles whatsoever.

However, the actual mistake in Nauenberg’s argument is the assumption, that via $\vec{p}=m{\vec{v}}_{B}$ the momentum of the particles should be constrained, i.e., that Bohm’s theory has not only a “hidden variable” for the particle position, but also for momentum (and why stop here and not add energy, spin, angular momentum etc.—as indicated above). That this cannot be the case is acknowledged by all scientists working on the field. Technically speaking this follows from the Kochen–Specker no-go theorem which implies that such a scheme contradicts quantum theory [12]. Now, Bohm’s theory avoids this problem by not introducing such additional variables for momentum, spin and the like. Reference [4] re-introduces them in the case of momentum (or rather velocity) and demonstrates (correctly) the inconsistency of this *modified* theory.

However, all this still leaves open how one should actually think about these quantities within Bohm’s theory. The starting point is the observation that any measurement of, say, momentum or spin involves a position measurement. The momentum of a charged particle is usually inferred from the bending inside a homogeneous magnetic field or the spin from the position measurement after the particle passing a Stern–Gerlach magnet. The outcome of these experiments is—according to Bohm—determined by the wave function and the initial *position*(*s*) and not by the value of any other “hidden variable”. This is expressed by saying that all quantities but position are “contextualized” (compare the discussion in [13]).

To see better what this means take the above example of a Stern–Gerlach experiment to determine the spin of a silver atom. Suppose that the north-pole of the magnet is situated above the south-pole and that a single silver atom gets deflected up (call this deflection towards the north pole “spin up”). The reason for this specific outcome lies—according to Bohm’s interpretation—in the fact that the initial position of the coordinate was above the symmetry plane of the system. Thus, a reversed orientation of north- and south-pole would have led to an up deflection still while this time (“deflection towards the south pole”) the opposite spin (“down”) would have been assigned to the atom. Note, that this example illustrates also *in nuce* how the Bohm interpretation explains definite outcomes for each single measurement, hence, solves the infamous measurement problem.

In other words, according to this view the specific spin value is not an intrinsic property of the particle but depends on the wave function, ψ, the configuration, $q_{i}$, and the experimental arrangement (viz. the “context”) as well. The same holds for the momentum, energy etc.

All this illustrates that the “Bohmian particle” should not be confused with, say, an electron. The latter is a fermion with specific mass and charge. In addition the “measurement” of momentum, energy and the like gives certain results which can be predicted by the theory (the term “measurement” has been put into scare quotes since it does not reveal a preexisting value of these quantities). The former just has the properties “position”. To describe the “electron” the de Broglie–Bohm interpretation needs both, the configuration *and* the wave function. In Daumer et al. this contextuality is even put into a wider context and the authors argue against what they call “naive realism about operators”. They conclude [14]:

“We thus believe that contextuality reflects little more than the rather obvious observation that the result of an experiment should depend upon how it is performed!”

## 5. Summary

We have dealt with the common misconception, that the de Broglie–Bohm theory does not only assign position to each quantum object but also a well defined momentum (or the value of any other observable). This idea is apparently very reasonable since the velocity of the Bohm-particles can certainly be multiplied with its mass. Michael Nauenberg has turned this misconception into an ingenious argument against the dBB theory—or rather into an argument against this modified version of it.

However, within the de Broglie–Bohm theory the product of velocity and mass has no physical meaning. In general its ambition is not to restore a classical world-view, but to solve the conceptual problems of quantum theory. In doing so any system is described by the pair of wave function and configuration. This implies an interesting reinterpretation of the property-concept. Properties like mass, charge, spin or momentum cannot be assigned to the object moving along the well-defined trajectories. Instead, they “belong” to the wave function, or rather: the result of a measurement of these quantities is determined by the wave function, the configuration and the specific experimental arrangement and does not reveal a previously existing (“hidden”) value. On the level of the individual trajectories concepts like momentum, energy and the like lose their meaning. This should not confuse anybody, since this motion is ruled by Bohmian mechanics and not by Newtonian mechanics.

All this may appear odd and nobody has to like or even to support the Bohm interpretation. However, it has to be acknowledged that it is not only a consistent interpretation of quantum mechanics but includes also “quantum weirdness”—like any other interpretation of quantum theory.
